# Intraoral and facial rehabilitation retained with zygomatic implants and magnets after complete resection of the maxilla, lip and nose: A clinical report

**DOI:** 10.4317/jced.60659

**Published:** 2023-08-01

**Authors:** Elizabeth Alfenas, Isadora Silva, Davidson Oliveira, Paul Tanner, Francisca Jardilino, Cláudia Bhering, Amália Moreno

**Affiliations:** 1 Adjunct Professor, Department of Oral Surgery, Pathology, and Clinical Dentistry; School of Dentistry, Federal University of Minas Gerais (UFMG), Belo Horizonte, MG, Brazil; 2 Postgraduate student, Department of Oral Surgery, Pathology, and Clinical Dentistry; School of Dentistry, Federal University of Minas Gerais (UFMG), Belo Horizonte, MG, Brazil; 3 Maxillofacial surgeon, Private Dental Clinic, Belo Horizonte, Brazil; 4 Adjunct Professor, Prosthetics Services, Huntsman Cancer Institute, University of Utah, Salt Lake City, UT, United States; 5 Adjunct Professor, Department of Restorative Dentistry; School of Dentistry, Federal University of Minas Gerais (UFMG), Belo Horizonte, MG, Brazil

## Abstract

Prosthetic rehabilitation is an option available for patients with extensive maxillofacial defects with the ability to restore esthetics and function. The surgical procedure can result in anatomical and functional sequelae leading to functional, psychological and aesthetic disorders. This clinical case report describes the unique and highly-specialized fabrication method of an oral and facial prosthesis for a patient with a near total maxillectomy, excision of upper lip, rhinectomy and radiotherapy for treatment of an extensive malignant neoplasm. Four carefully placed zygomatic implants were used to retain an oral obturator and an external mid-facial prosthesis. A well-functioning maxillofacial prosthesis is essential for improving quality of life, psychological adjustment to cancer and cancer related disabilities, and a positive coping response. The prosthetic rehabilitation allowed the patient’s reintegration into society accompanying the satisfactory restoration of aesthetics, phonetics, mastication, and deglutition.

** Key words:**Maxillectomy, rhinectomy, maxillofacial prosthesis, zygomatic implants, head and neck neoplasm.

## Introduction

Oral cavity cancer is one of the most common malignancies, especially in developing countries (1). When it affects the middle third of the face, it can include different structures and, depending on its extension, result in significant disfigurement (2).

The surgical procedure can result in anatomical and functional sequelae leading to functional, psychological and aesthetic disorders (3,4). The resultant functional disabilities include nasal emission during speech causing hypernasality and altered speech intelligibility and incompetent swallowing followed by nasal regurgitation of food and fluids, both leading to psychosocial morbidity and impaired global quality of life (QOL) (5). Individuals with deformities usually can have a loss of identity due to face misrecognition (2).

The functional and aesthetic rehabilitation of these patients is one of the most significant challenges for the multidisciplinary team (6). The oral and maxillofacial prosthesis promotes the restoration of lost stomatognathic and craniofacial structures, rehabilitating compromised aesthetics and functions (chewing, swallowing and phonetics) (3,7-9).

Using osseointegrative implants for a prosthesis in the maxillofacial region has proven to be a reliable treatment option with high long-term success rates, promoting excellent retention and stability (3,4). Although controversial, rehabilitation with implants after radiotherapy is a viable treatment option, in addition to often being the only possibility of rehabilitation for the patient with an extensive surgical defect. Whilst the short to medium term implant survival in these cases is high, multiple factors require careful consideration for a favorable outcome (10). The zygomatic implant was designed in 1998 specifically for use in compromised bone, including severe atrophy, congenital disabilities, and tumor resection defects (11). This clinical case report describes the challenging rehabilitation of a significant facial defect using a sectioned maxillofacial prosthesis that includes an intraoral obturator and an extraoral facial prosthesis completely retained by zygomatic implants after radiotherapy.

## Case Report

A 56-year-old-man, stopped smoking before surgery, with a medical history of resection of tumor of the midface due to treatment of invasive basal cell carcinoma, history of radiotherapy and enteral diet via tube. The patient received treatment from the Oral and Maxillofacial Prosthesis Clinic at the Federal University of Minas Gerais (UFMG) in Brazil for oral and facial prosthetic rehabilitation. Due to treatment, the patient presented with an extensive maxillary defect involving the absence of most of the hard palate, the upper lip, the nose and nasal cavity. There was only preservation of the maxilla in the regions of bilateral maxillary tuberosities and soft palate. The patient was unable to speak, chew, swallow, and felt extremely reluctant to interact with people outside of his home. Quality of life for this patient was very low for this patient after cancer treatments.

The proposed treatment plan was the construction of a combined sectional prosthesis, including an oral obturator retained by zygomatic implants, since there would be no other possibility of retaining the palatal obturator prosthesis. A mid-facial prosthesis could then be attached to the oral obturator with stainless steel encased magnets. A 3D printed model generated from computed tomography images of the cranium aided in the planning and insertion angle of the zygomatic implants. The application used for generating the 3D models was InVesalius by the CTI Renato Archer in Campinas, Brazil. This process is too lengthy to describe in this article.

**Figure 1:**
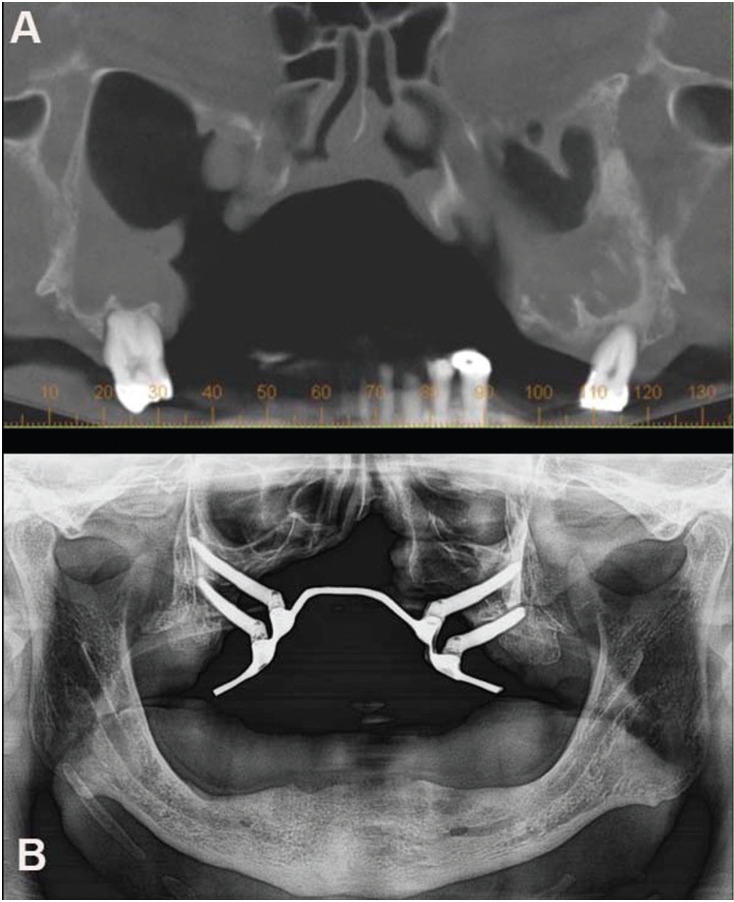
A: Planning for locating the implants in the zygomatic bone region. B: Radiological examination after implants surgery in the zygomatic bone.

After planning and fabrication of a surgical guide, four zygomatic implants with an external hexagon platform (SIN; SIN Implant System) were placed: 4.5 x 40 mm (right canine region), 4.5 x 35 mm (right first molar region), 4.5 x 32.5 mm (left canine region) and 4.5 x 40 mm (left first molar region) (Fig. 1). At the end of the surgery, prosthetic components Zybutment Standard (SIN; SIN Implant System) 4.5 x 3 mm were attached to each of the implants. The first immediate impression was taken using the addition-cure silicone (Elite HD+; Zhemarck) by the open tray technique with transfers for mini-abutments (SIN; SIN Implant System) in position for transferring the placement of the implants and making a titanium palatal bar. The titanium palatine bar screwed onto the prosthetic intermediates of the zygomatic implants was installed 48 hours after surgery to support and retain the palatal obturator prosthesis.

**Figure 2:**
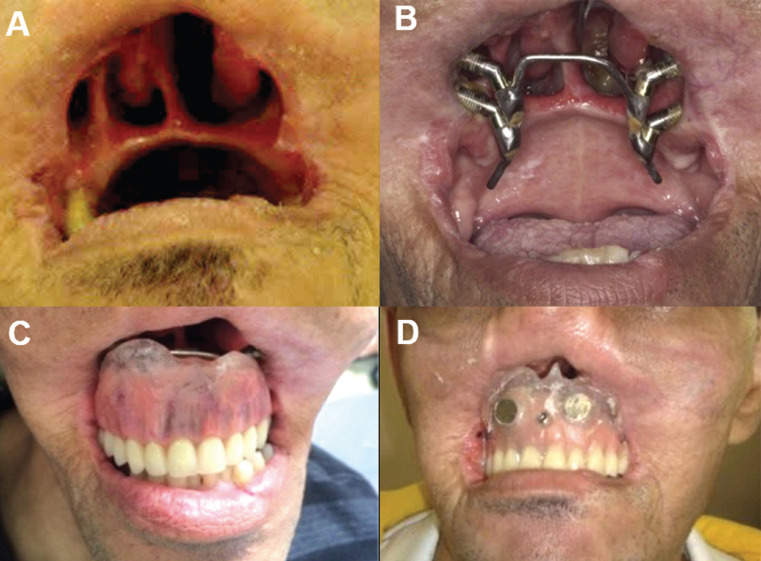
A: Initial clinical examination of the patient. B: Palatal titanium bar screwed into implants. C: Palatal obturator prosthesis retained on the titanium bar. D: Palatal obturator prosthesis retained on a palatal titanium bar with two magnets and an attachment in the intraoral.

A subsequent impression including the palatine bar was obtained with addition-cure silicone for making the palatal obturator prosthesis (Elite HD+; Zhemarck). From the working model, the test base and the wax plan were made to guide the assembly of the teeth. After defining and selecting the teeth, the prosthesis was polymerized with Polymethylmethacrylate (PMMA). Following the fabrication of the oral obturator, two stainless steel encased magnets and a ball-type attachment were polymerized in the obturator prosthesis with PMMA to support and retain the mid-facial prosthesis (Fig. 2).

**Figure 3:**
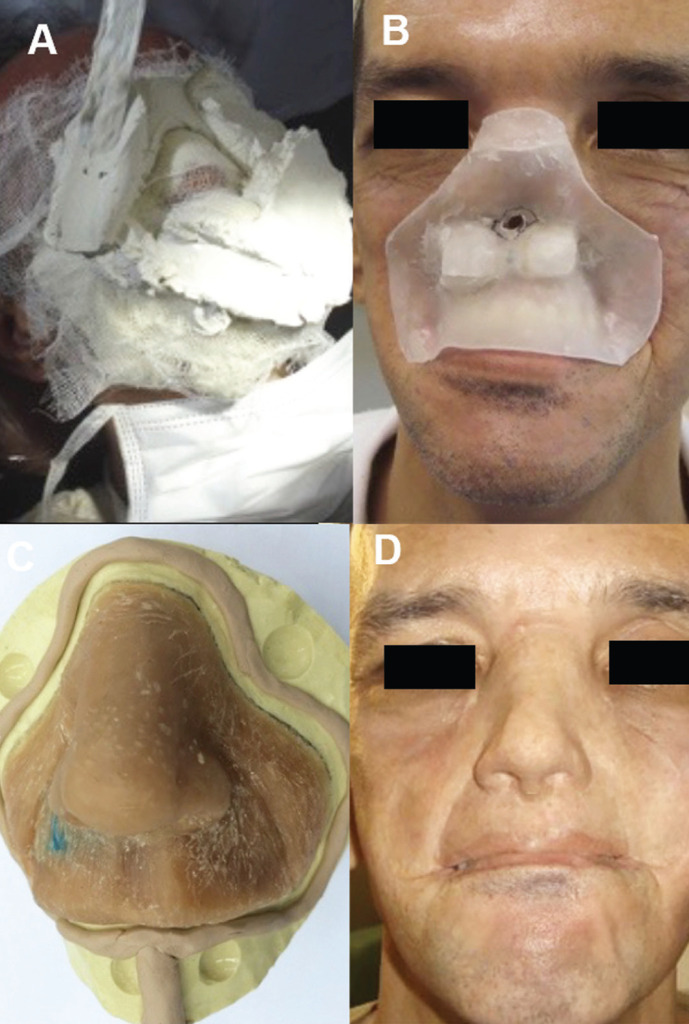
A: Molding of the patient’s face for making a maxillofacial prosthesis. B: Waxing of the maxillofacial prosthesis base. C: Sculpture of the maxillofacial prosthesis. D: Maxillofacial prosthesis installed on the patient and retained over the palatal obturator prosthesis.

After proving the proper and adequate fit of the oral obturator, the polar magnets were placed on the obturator and, a new impression of the magnets and ball attachment was made with addition-cure silicone (Elite HD+; Zhemarck). Leaving this silicone impression in place, another impression of the face was obtained with irreversible hydrocolloid (Hydrogum 4; Zhemarck) material while protecting the airway of the patient with gauze. The patient sat in an upright position and bit on a tongue depressor in order to confine breathing to the mouth. This is important so that no impression material enters the airway through the nasal passage. The facial impression was used to obtain the plaster model (Durone IV; Dentsply Sirona) of the face of the patient and to index the magnets and ball attachment using the silicone impression of those attachments. A wax-up of the nose and upper lip was made and fitted to the patient. A stone mold was made of the wax-up and the facial prosthesis was made by pressing a medical-grade silicone elastomer (Silastic Q7-4735; Silastic). External pigmentation using a dispersed version of the same silicone while mixed with the appropriate colored pigments, camouflaged the prosthesis (Fig. 3). After installing the oral obturator and mid-facial prosthesis, the patient learned about placement, removal, and hygiene. The patient reported extreme satisfaction with the aesthetic result and effectiveness in chewing, speaking and swallowing, providing his reintegration into society and return to community activities.

## Discussion

The restoration of radical facial defects is a challenge for specialists in oral and maxillofacial prosthetics. In these patients, a prosthesis composed of two distinct parts should be considered, as it is an even more significant challenge to achieve prosthesis retention (8).

The use of implants as part of rehabilitation in cancer patients with maxillofacial resections has become common to improve prosthesis retention, which directly influences patient acceptance (3,12). Chang *et al*. using a questionnaire with 28 questions addressing the subject’s perception of the oral and maxillofacial prosthesis, it was generally observed that individuals with implant-retained prosthesis were significantly more likely to have positive responses about ease of placement and removal and reliability of retention during various activities than those with adhesive-retained prosthesis (3).

Implants are essential for better results of stability, support and retention of large mid-facial defects, which promotes a direct improvement in masticatory function and phonation, having a substantial and positive impact on their quality of life (13). In the present study, support and retention of maxillofacial prosthesis were only possible through zygomatic implants. Through them, it was possible to retain the oral obturator, which, with magnets and a ball attachment, could connect and retain the facial prosthesis. Therefore, it is necessary to build the prosthesis in two separate parts so that there is retention and rehabilitation of the masticatory function, deglutition, phonation, and aesthetics.

The installation of implants in patients undergoing radiotherapy is widely discussed and controversial in the literature. The success rate of implants placed in irradiated patients is slightly lower, although not significant, than in non-irradiated patients (6). However, patients undergoing extensive oral and maxillofacial surgeries and radiotherapy are good candidates for zygomatic implants despite the increased risk of implant failure (10).

Where such cases are rare and statistically significant treatment outcomes are not standardized, clinical assessment of treatment modalities should include outcomes of other patients not only survival rates but also specific measures that reflect patient well-being and adaptation to illness and illness related disabilities (14). A well-functioning maxillofacial prosthesis is essential for improving quality of life, psychological adjustment to cancer and cancer related disabilities, and a positive coping response (14).
